# Supplementary material for: Learning to Hunt on the Go: Dietary Changes During Development of Rhinolophid Bats
Source: Animals (Basel). 2024 Nov 16;14(22):3303. doi: 10.3390/ani14223303 (PMC11591299; doi:10.3390/ani14223303)
Supplement: Supplementary file 1 [file animals-14-03303-s001.zip › animals-3285553-SuppInfo1_Development_analysis.pdf]

Learning To Hunt On The Go: dietary changes during development of rhinolophids bats.

Behavioral Ecology and Sociobiology

Miren Aldasoro[1]*, Nerea Vallejo[1], Lander Olasagasti[1], Oihane Diaz de Cerio[1] & Joxerra Aihartza[1]

[1] *Department of Zoology and Animal Cell Biology, University of the Basque Country UPV/EHU, Sarriena s/n, 48940, Leioa, The Basque Country, Spain*

miren.aldasoro@ehu.eus

Supporting Information 1:

Development analysis of horseshoe bats: Methods and results

The epiphyseal gap (EG) was measured on the photographs (in millimetres) (Adobe Photoshop 2019, Adobe Inc.) by summing the two observable —translucent— cartilage bands corresponding to the proximal and distal epiphyseal or growth plates (based on Kunz and Anthony 1982) (Fig. S1).

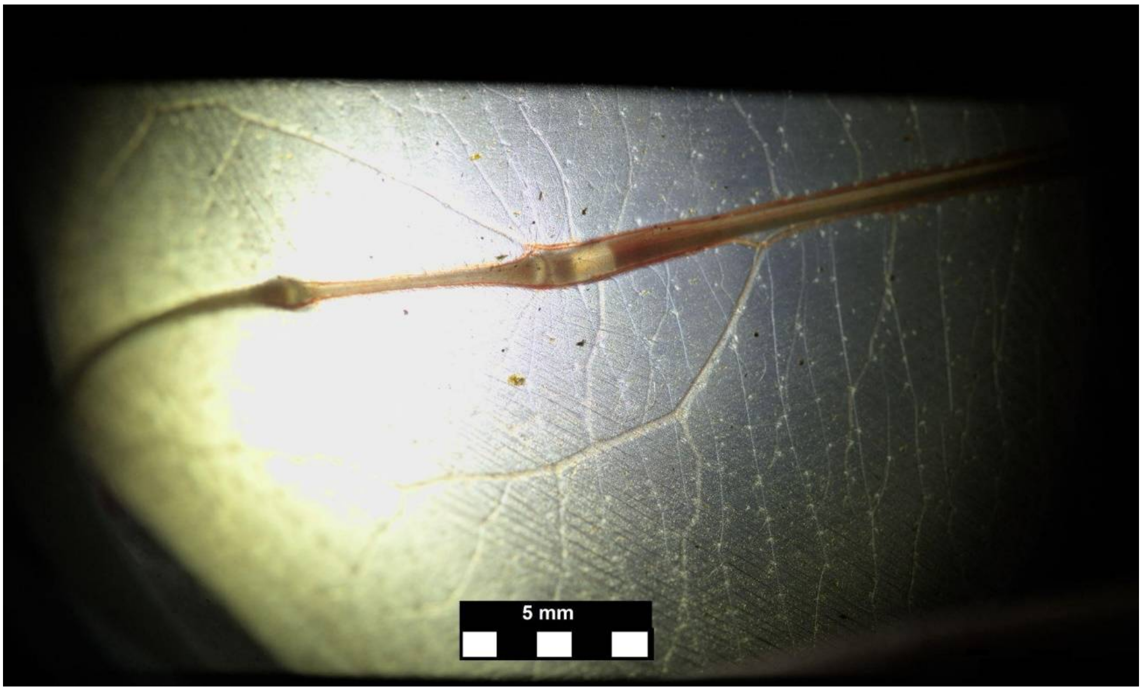

Figure S1. Epiphysis of a young individual. The observable —translucent— cartilage bands corresponding to the proximal and distal epiphyseal or growth plates. The epiphyseal gap (EG) was calculated by summing these two measures.

For each bat species, Pearson's correlation test was held for resting frequency (RF), forearm length (FL), weight (WG) and epiphyseal gap (EG). The p-value threshold (α) was adjusted by the Bonferroni correction (α/m). As we conducted 6 correlations for each species (m=6), the threshold was established in 0.8 %. The effect of sex upon these parameters was also analysed using the T-student test, setting sex as a fixed variable, using function *t.test* for R version 4.2.0 (R Core Team, 2022).

Our results showed that development follows different allometric patterns in each studied horseshoe bat species (Table 1). Sex differences were only observed in *R. hippposideros*. In this case, the T-test proved sex differences were found in parameters FL and RF (t_{FA} = 4.025, p = 0,001; t_{RF} = 6.358 p < 0.001), as females show higher FL and RF. Thus, correlations were calculated separately for males and females (Table 1b). Males did not show any significant correlation for any of the developmental traits, whereas in the case of females, WG and EG were negatively correlated.

In the case of *R. euryale* and *R. ferrumequinum* T-test did not reveal any significant differences between males and females. Therefore, all individuals were considered together for the correlation analysis. For *R. euryale,* after the Bonferroni correction no significant correlations were found. Finally, in *R. ferrumequinum,* even though RF and FL do not significantly correlated with each other, both traits did correlate negatively with EG and WG separately (Table 1a).

Table S1a. Correlation between development parameters for *R. ferrumequinum* (n=29) and *R. euryale* (n=25). (RF: Resting frequency; WG: Weight; FL: Forearm Length; EG: Epiphyseal gap). R: The Pearson correlation coefficient. Significant correlations in bold (p-value < 0.008).

	R. ferrumequinum			*R. euryale*		
	**RF**	**WG**	**EG**	**RF**	**WG**	**EG**
FL	R = 0.333<br>p = 0.104	**R = 0.643<br>p = 0.001**	**R = -0.663<br>p = 0.001**	R = 0.044<br>p = 0.833	R = 0.057<br>p = 0.578	R = 0.117<br>p = 0.001
RF		**R = 0.535<br>p= 0.006**	**R = -0.573<br>p = 0.003**		R = -0.217<br>p = 0.297	R = -0.564<br>p = 0.03
WG			R = - 0.367<br>p = 0.071			R = 0.107<br>p = 0.612

Table S1b. Correlation between development parameters for *R. hipposideros* females (n=13) and males (n=11). (RF: Resting frequency; WG: Weight; FL: Forearm Length; EG: Epiphyseal gap). Significant correlations in bold.

	R. hipposideros (Females)			*R. hipposideros (Males)*		
	**RF**	**WG**	**EG**	**RF**	**WG**	**EG**
FL	R = 0.369<br>P = 0.214	R = 0.529<br>P = 0.063	R = -0.449<br>P = 0.124	R =-0.412<br>P = 0.208	R = 0.237<br>P = 0.483	R = -0.210<br>P = 0.536
RF		R = 0.274<br>P = 0.364	R = -0.056<br>P = 0.855		R = 0.031<br>P = 0.929	R = -0.264<br>P = 0.433
WG			**R = - 0.842**<br>**P = 0.0001**			R = -0.264<br>P = 0.433

Finally, we conducted a separate T-test to the adult individuals of each bat species for the parametres WG and FL setting sex as a fixed variable, in order to discover any sexual dimorphism as found in juvenile *R. hipposideros.* We found that females of the three species had significantly longer forearms (FL), even if for *R. ferrumequinum* the sample size was too small to be statistically significant (n=3) (Table 2).

Table 2. Results of the T-test for the mean values of the variables Weight (WG) and Forearm Length (FL) of adult individuals with sex as the fixed variable. NF: Female sample size; NM: Male sample size; T: T value; P: p-value. Significant values are in bold.

	Sample size	WG	FL
R. euryale	N_F = 9<br>N_M = 11	T = 2.080<br>P = 0.052	**T = 3.340**<br>**P = 0.004**
R. hipposideros	N_F = 13<br>N_M = 8	T = 1.178<br>P = 0.254	**T = 2.154**<br>**P = 0.044**
R. ferrumequinun	N_F = 24<br>N_M = 3	T = 1.839<br>P = 0.078	**T = 3.139**<br>**P = 0.017**
