# Supplementary material for: Learning to Hunt on the Go: Dietary Changes During Development of Rhinolophid Bats
Source: Animals (Basel). 2024 Nov 16;14(22):3303. doi: 10.3390/ani14223303 (PMC11591299; doi:10.3390/ani14223303)
Supplement: Supplementary file 1 [file animals-14-03303-s001.zip › SuppInfo3_RLQ_4thCorner.pdf]

Learning To Hunt On The Go: dietary changes during development of rhinolophids bats.

Behavioral Ecology and Sociobiology

Miren Aldasoro[1]*, Nerea Vallejo[1], Lander Olasagasti[1], Oihane Diaz de Cerio[1] & Joxerra Aihartza[1]

[1] *Department of Zoology and Animal Cell Biology, University of the Basque Country UPV/EHU, Sarriena s/n, 48940, Leioa, The Basque Country, Spain*

miren.aldasoro@ehu.eus

Supporting Information 3:

RLQ and 4th corner analysis for the predator-prey trait based analysis

Methodology

Briefly, the three matrices are: (1) the *R matrix* (m x n) is a bat individual vs bats' characteristics table, which describes the "environment" of the (n) sampled bats according to a set of (m) individual traits. (2), the *Q matrix* (p x s) is a prey vs prey's trait table, where (s) morphological and functional traits describe (p) prey arthropod species. (3), the *L matrix* (n x p), which connects the first two matrices by describing the ecological interactions between bats and prey. It consists of a matrix of the (p) prey species within the diet of the (n) bat individuals, measured as the weighted percentage of occurrence of each prey. Based on the matrixes, the RLQ analysis builds a new matrix that relates the bat characteristics to the prey traits. This last is used to perform a standard PCA-like eigendecomposition. Finally, we applied a fourth-corner analysis directly on the results of the RLQ analysis to summarise and test the significance of the associations between the RLQ axes and the functional traits of bats and prey.

Separate ordinations of each matrix are required before an RLQ analysis. Thus, a Correspondence Analysis (CA) was applied to the matrices R (bats traits), Q (prey traits) and L (diet), and the matrices were ordered by a Hill-Smith analysis since they contain both qualitative and quantitative variables. Graphical outputs of the RLQ analysis were used to summarise the central relationships between the functional traits of bats and moths. As the missing values prevented a proper analysis, these were replaced by the median values for the given class of the traits for each matrix to prevent data deviation (Acuña & Rodriguez, 2004).

Results

Rhinolophus euryale

Table S1. The combination of fourth-corner and RLQ results in the analysis of the diet of *R. euryale* individuals (see Figure 5): (A) fourth-corner tests between the first two RLQ axes for bat traits (AxQ1/AxQ2) versus prey traits; (B) fourth-corner tests between the first two RLQ axes prey traits (AxR1/AxR2) versus bat traits.

(A) Bat traits	Axis Q1	Axis Q2	(B) Moth traits	Axis R1	Axis R2
**Sex**	0.9739	0.9157	**Hardness**	0.0075	0.6776
**Age**	0.0006	0.9157	**Volume**	0.0012	0.6776
**Weight**	0.0024	0.9157	**Wing Loading**	0.6776	0.3064
**Forearm Length**	0.0024	0.9157			
**Resting Frequency**	0.0008	0.6906			
**Epiphyseal Gap**	0.0006	0.9157			

NOTE: Significant ($p < 0.05$) positive associations are represented by dark grey cells and negative associations by light grey cells. p-values are given in each cell.

Table S2. Summary of fourth-corner results in the analysis of the diet of *R. euryale* individuals. WG: Weight; FL: Forearm Length; RF: Resting Frequency; EG: Epiphyseal Gap.

	Sex	Age	WG	FL	RF	EG
**Hardness**	0.8201	0.0036	0.0308	0.0328	0.0021	0.0021
**Volume**	0.9220	0.0009	0.0012	0.0021	0.0021	0.0009
**Wing Loading**	0.9377	0.9377	0.9190	0.7749	0.4071	0.9190

NOTE: Significant ($p < 0.05$) positive associations are represented by dark grey cells and negative associations by light grey cells. p-values are given in each cell.

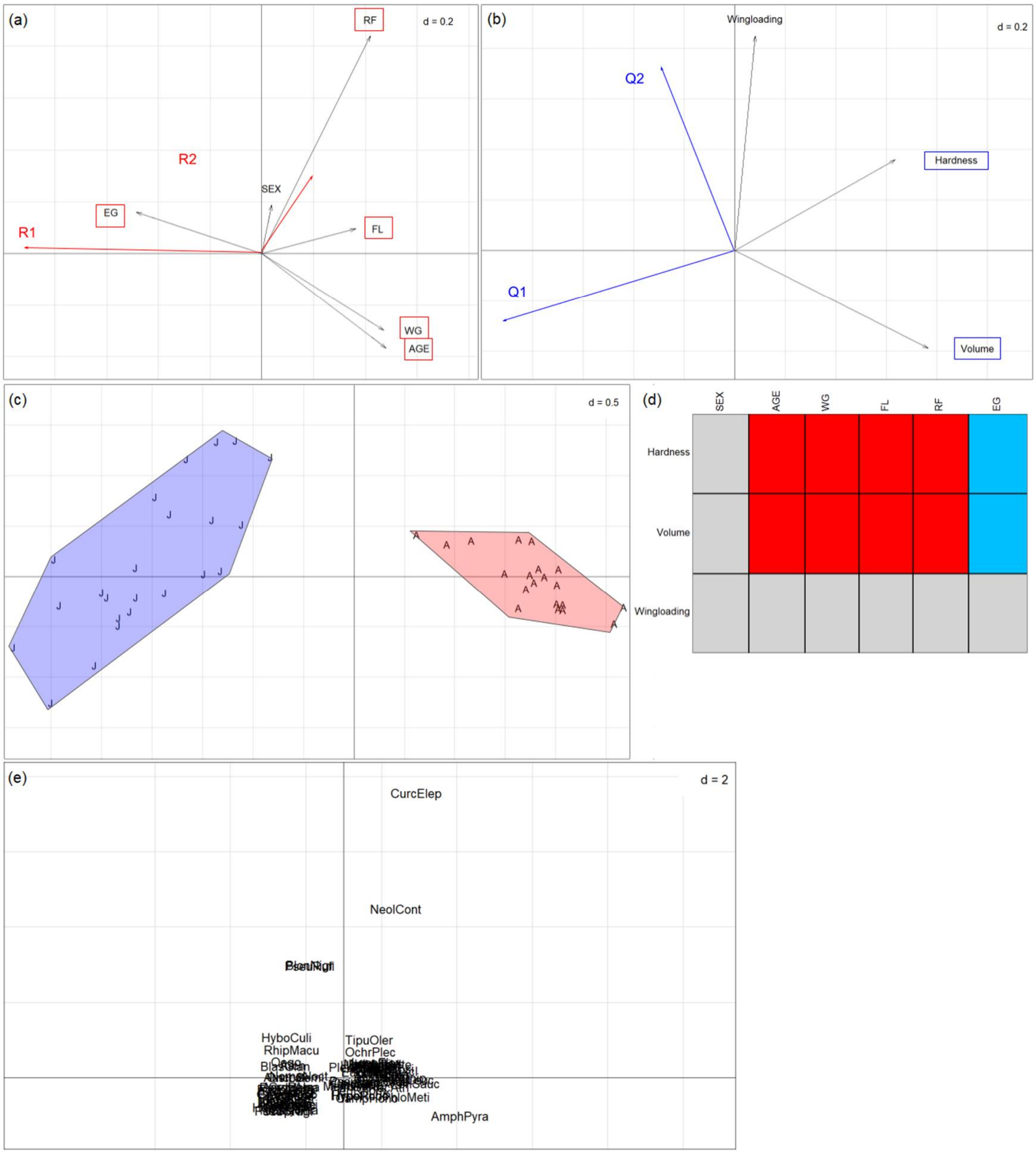

Figure S1. Results of RLQ analyses for the diet of *R. euryale* individuals: (a) coefficients for bat traits (EG: Epiphyseal Gap; FL: Forearm Length; RF: Resting Frequency; WG: Weight), (b) coefficients for prey traits (traits framed in a rectangle represent a significant association with the axes), (c) eigenvalues and scores of bat individuals (Red A: Adults; Blue J: Juveniles), (d) results of the 4th corner analysis and (e) eigenvalues and scores of prey species. Red cells represent positive associations and blue cells represent negative ones (p-value < 0.05). Panels display the first two axes only, with d-values referring to grid size. Codes for prey species are available in Supporting Table S2. Monte-Carlo test: observed statistic = 1.793 (standardized observed statistic = 5.678), with p-value < 0.0001.

Rhinolophus hipposideros

Table S3. The combination of fourth-corner and RLQ results in the analysis of the diet of *R. hipposideros* individuals (see Figure 6): (A) fourth-corner tests between the first two RLQ axes for bat traits (AxQ1/AxQ2) versus prey traits; (B) fourth-corner tests between the first two RLQ axes prey traits (AxR1/AxR2) versus bat traits.

(A) Bat traits	Axis Q1	Axis Q2	(B) Moth traits	Axis R1	Axis R2
**Sex**	0.0388	0.5673	**Hardness**	0.0687	0.9075
**Age**	0.0308	0.8692	**Volume**	0.0318	0.4746
**Weight**	0.0321	0.6265	**Wing Loading**	0.0842	0.30915
**Forearm Length**	0.5673	0.3470			
**Resting Frequency**	0.024	0.6940			
**Epiphyseal gap**	0.0321	0.6940			

NOTE: Positive associations are represented by dark grey cells and negative associations by light grey cells ($p < 0.05$). p-values are given in each cell.

Table S4. Summary of fourth-corner results in the analysis of the diet of *R. hipposideros* individuals. WG: Weight; FL: Forearm Length; RF: Resting Frequency; EG: Epiphyseal Gap.

	Sex	Age	WG	FL	RF	EG
**Hardness**	0.0529	0.0681	0.0681	0.4753	0.0450	0.0937
**Volume**	0.0270	0.0450	0.0937	0.1268	0.0198	0.0675
**Wing Loading**	0.4078	0.0680	0.0624	0.7902	0.1171	0.0675

NOTE: Significant ($p < 0.05$) positive associations are represented by dark grey cells and negative associations by light grey cells. p-values are given in each cell.

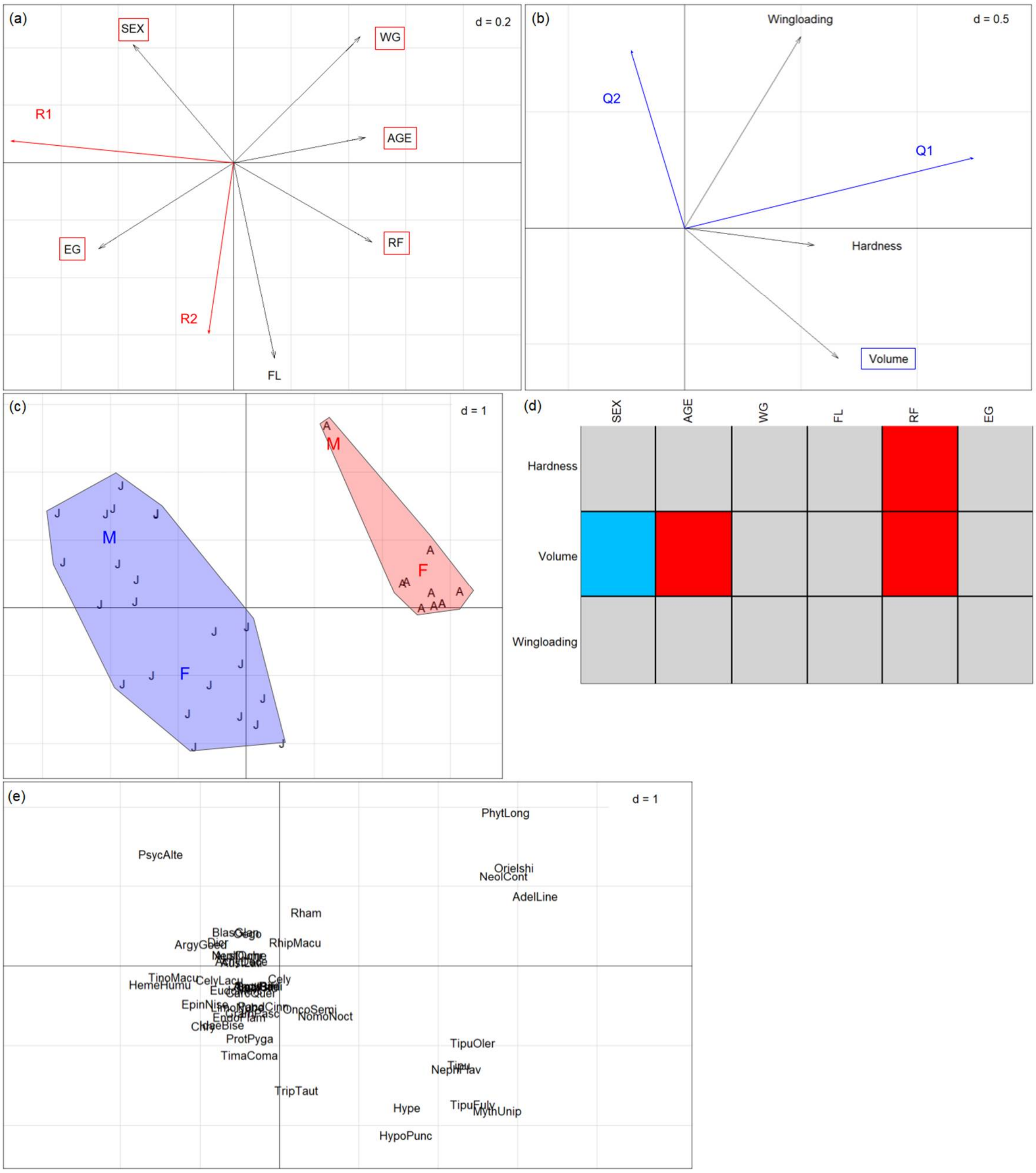

Figure S2. Results of RLQ analyses for the diet of *R. hipposideros* individuals: (a) coefficients for bat traits (EG: Epiphyseal Gap; FL: Forearm Length; RF: Resting Frequency; WG: Weight), (b) coefficients for prey traits (traits framed in a rectangle with dotted lines represent an association with the axes ($p < 0.1$)), (c) eigenvalues and scores of bat individuals (Red A: Adults; Blue J: Juveniles; M: Males; F: Females), (d) results of the 4th corner analysis and (e) eigenvalues and scores of prey species. Red cells represent positive associations and blue cells represent negative ones (p-value < 0.05). Panels display the first two axes only, with d-values referring to grid size. Codes for prey species are available in Supporting Table S2. Monte-Carlo test: observed statistic = 0.550 (standardized observed statistic = 3.460), with p-value < 0.01.

Rhinolophus ferrumequinum

Table S5. The combination of fourth-corner and RLQ results in the analysis of the diet of *R. ferrumequinum* individuals: (A) fourth-corner tests between the first two RLQ axes for bat traits (AxQ1/AxQ2) versus prey traits; (B) fourth-corner tests between the first two RLQ axes prey traits (AxR1/AxR2) versus bat traits.

**(A) Bat traits**	**Axis Q1**	**Axis Q2**	**(B) Moth traits**	**Axis R1**	**Axis R2**
**Sex**	0.6580	0.3914	**Hardness**	0.0839	0.8876
**Age**	0.3633	0.9647	**Volume**	0.5720	0.5126
**Weight**	0.7413	0.8926	**Wing Loading**	0.1652	0.6512
**Forearm Length**	0.4935	0.8375			
**Resting Frequency**	0.0881	0.9968			
**Epiphyseal gap**	0.1805	0.8544			

NOTE: Positive associations are represented by dark grey cells and negative associations by light grey cells ($p < 0.1$). p-values are given in each cell.

Table S6. Summary of fourth-corner results in the analysis of the diet of *R. ferrumequinum* individuals. WG: Weight; FL: Forearm Length; RF: Resting Frequency; EG: Epiphyseal Gap.

	**Sex**	**Age**	**WG**	**FL**	**RF**	**EG**
**Hardness**	0.87504	0.80145	0.87504	0.87504	0.7896	0.7896
**Volume**	0.87504	0.9101	0.9101	0.87504	0.87504	0.87504
**Wing Loading**	0.87504	0.87504	0.9101	0.87504	0.7896	0.86976

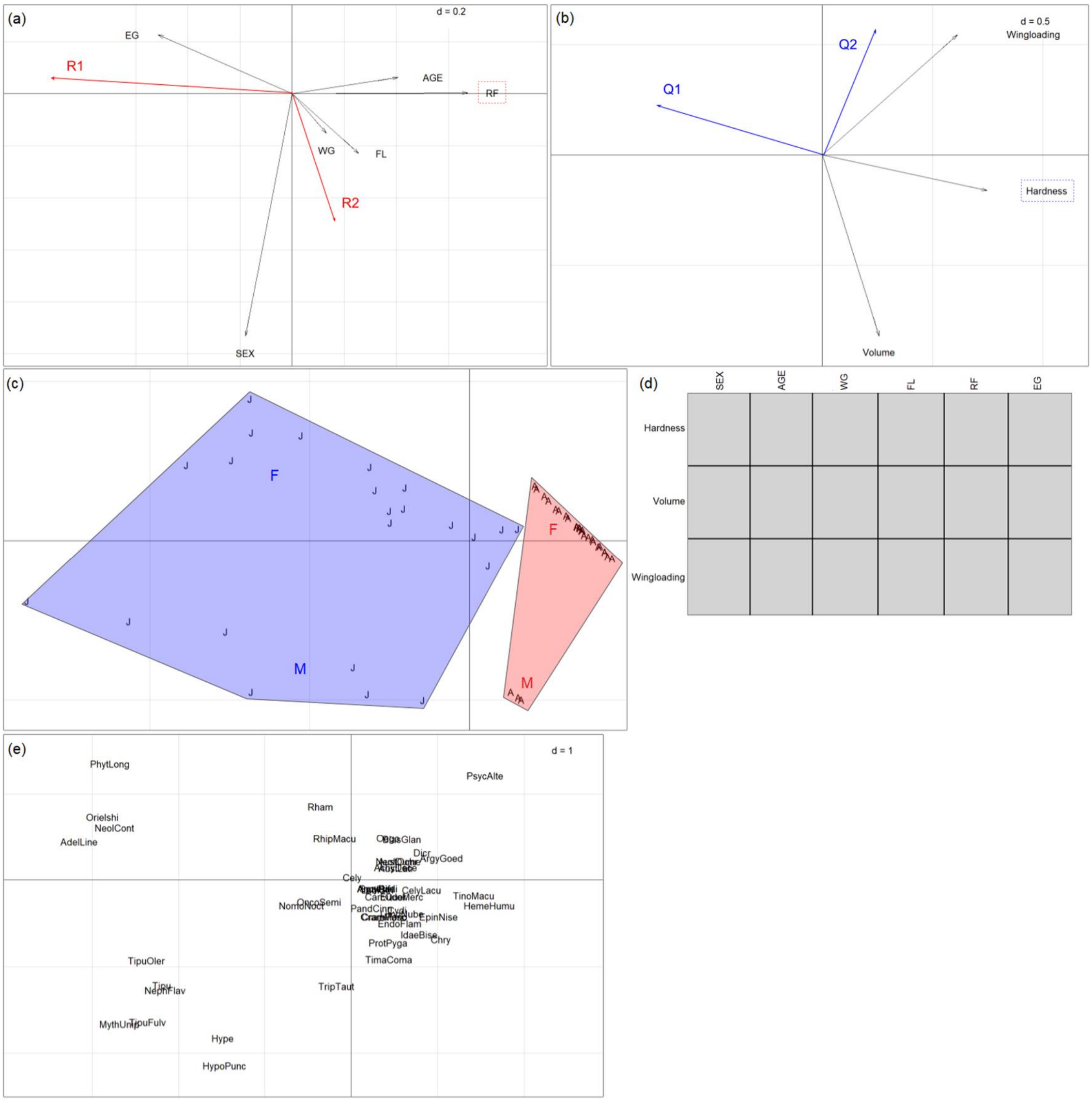

Figure S3. Results of RLQ analyses for the diet of *R. ferrumequinum* individuals: (a) coefficients for bat traits (EG: Epiphyseal Gap; FL: Forearm Length; RF: Resting Frequency; WG: Weight), (b) coefficients for prey traits (traits framed with dotted lines in a rectangle represent an association with the axes ($p < 0.1$)), (c) eigenvalues and scores of bat individuals (Red A: Adults; Blue J: Juveniles; M: Males; F: Females) and (d) results of the 4th corner analysis and (e) eigenvalues and scores of prey species. Red cells represent positive associations and blue cells represent negative ones (p-value < 0.05). Panels display the first two axes only, with d-values referring to grid size. Codes for prey species are available in Supporting Table S2. Monte-Carlo test: observed statistic = 0.054 (standardized observed statistic = -0.175), with p-value 0.451.
